# fastMRI+, Clinical pathology annotations for knee and brain fully sampled magnetic resonance imaging data

**DOI:** 10.1038/s41597-022-01255-z

**Published:** 2022-04-05

**Authors:** Ruiyang Zhao, Burhaneddin Yaman, Yuxin Zhang, Russell Stewart, Austin Dixon, Florian Knoll, Zhengnan Huang, Yvonne W. Lui, Michael S. Hansen, Matthew P. Lungren

**Affiliations:** 1 Microsoft Research, Redmond, USA; 2 University of Wisconsin-Madison, Department of Radiology, Madison, USA; 3 University of Wisconsin-Madison, Department of Medical Physics, Madison, USA; 4 University of Minnesota, Department of Electrical and Computer Engineering, Minneapolis, USA; 5 Stanford University, School of Medicine, Stanford, USA; 6 Duke University, School of Medicine, Durham, USA; 7 New York University, School of Medicine, New York, USA

**Keywords:** Diagnostic markers, Diagnostic markers, Brain, Ligaments, Cartilage

## Abstract

Improving speed and image quality of Magnetic Resonance Imaging (MRI) using deep learning reconstruction is an active area of research. The fastMRI dataset contains large volumes of raw MRI data, which has enabled significant advances in this field. While the impact of the fastMRI dataset is unquestioned, the dataset currently lacks clinical expert pathology annotations, critical to addressing clinically relevant reconstruction frameworks and exploring important questions regarding rendering of specific pathology using such novel approaches. This work introduces fastMRI+, which consists of 16154 subspecialist expert bounding box annotations and 13 study-level labels for 22 different pathology categories on the fastMRI knee dataset, and 7570 subspecialist expert bounding box annotations and 643 study-level labels for 30 different pathology categories for the fastMRI brain dataset. The fastMRI+ dataset is open access and aims to support further research and advancement of medical imaging in MRI reconstruction and beyond.

## Abstract (summary)

**Table:** 

Measurement(s)	Pathotology annotations in knee and brain MRI images
Technology Type(s)	Expert delineation

## Background & Summary

Magnetic resonance imaging (MRI) is a widely utilized medical imaging modality critically important for a broad range of clinical diagnostic tasks including stroke, cancer, surgical planning, acute injuries, and more. Machine learning (ML) techniques have demonstrated opportunities to improve the MRI diagnostic workflow particularly in the image reconstruction task by saving time, reducing contrast, and leading in cases to FDA-cleared solutions^1–4^. Among the myriad applications of machine learning in medical imaging being explored, deep learning-based MRI reconstruction is showing considerable promise and is moving towards clinical impact.

ML-based MRI reconstruction approaches often require data from “raw” fully sampled k-space datasets in order to generate ground truth images. Public MRI datasets like Calgary-Campinas Public Dataset^5^, MRNet^6^, OAI^7^, SKM-TEA^8^, and mridata.org are available to empower ML-related research. Also, various datasets can be found in multiple medical image research challenges, including MC-MRREC and RealNoiseMRI. Most of these datasets only provided reconstructed MRI images (Note SKM-TEA dataset also provides knee tissue label and pathology detection information) or limited amount of raw data. Thus, large datasets of raw MRI measurements are generally not widely available. To address this need and facilitate cross-disciplinary research in accelerated MRI reconstruction using artificial intelligence, the fastMRI initiative was developed. fastMRI is a collaborative project between Facebook AI Research (FAIR), New York University (NYU) Grossman School of Medicine, and NYU Langone Health which includes the wide release of raw MRI data and image datasets^9^. While the fastMRI data has enabled exploration of ML-driven accelerated MRI reconstruction^10,11^, there is a lack of clinical pathology information to accompany the imaging data which has limited the reconstruction assessment approaches to validate quantitative metrics such as peak signal-to-noise ratio (pSNR)/structural similarity index measure (SSIM), leaving important questions regarding how various pathologies are represented in ML-based reconstruction unanswered^12^. For instance, low sensitivity and stability to clinically relevant features stall their clinical-aware applications^12–14^.

In this paper, we present wide availability of a complementary dataset of annotations, fastMRI+, consisting of human subspecialist expert clinical bounding box labelled pathology annotations for knee and brain MRI scans from the fastMRI multi-coil dataset: specifically encompassing 16154 bounding box annotations and 13 study-level labels for 22 different pathology categories on knee MRIs, as well as 7570 bounding box annotations and 643 study-level labels for 30 different pathology categories on brain MRIs. This new dataset is open and accessible to all for educational and research purposes with the intent to catalyse new avenues of clinically relevant, ML-based reconstruction approaches and evaluation.

## Methods

### MRI image dataset

The fastMRI dataset is an open-source dataset, which contains raw and DICOM data from MRI acquisitions of knees and brains, described in detail elsewhere^9^. The images used in this study were directly obtained from the fastMRI dataset, reconstructed from fully sampled, multi-coil k-space data (both knee and brain). The fastMRI dataset was managed and anonymized as part of a study approved by the NYU School of Medicine Institutional Review Board. Image reconstruction was performed by inverse Fast Fourier Transform of each individual coil and coil combination with root sum square (RSS) for the purpose of creating pre-annotation images in fastMRI+. The reconstructed images were subsequently converted to DICOM format for human expert reader (radiologist) annotation.

### Annotations

Annotation was performed using a commercial browser-based annotation platform (MD.ai, New York, NY) which allowed adjustment of brightness, contrast, and magnification of the images. Readers used personal computers to view and annotate the images using the mentioned annotation platform.

A subspecialist board certified musculoskeletal radiologist with 6 years in practice experience performed annotation for the knee dataset and a subspecialist board certified neuroradiologist with 2 years in practice experience performed annotation for the brain dataset. Annotation was performed with bounding box annotation to include the relevant label for a given pathology on a slice-by-slice level. When more than one pathology was identified in a single image slice, multiple bounding boxes were used.

All 1172 fastMRI knee MRI raw dataset studies were reconstructed and clinically annotated for fastMRI+. Each knee examination consisted of a single series (either proton density (PD) or T2-weighted) of coronal images where bounding box labels were placed on each slice where representative pathology was identified^15,16^. Effort was made to try to include all the pathology within the bounding box while limiting the normal surrounding anatomy. If the examination contained significant clinically limiting artifacts, then the annotation for “Artifact” was added as a study-level label. In these instances, an interpolation tool was used in which the first and last slice were each labelled and the user interface interpolated the labels on intervening slices. If no relevant pathology was identified on an examination, no labels were provided.

A sub selection of 1001 out of 5847 fastMRI brain MRI raw dataset studies were selected randomly for annotation. Each brain examination included a single axial series (either T2-weighted FLAIR, T1-weighted without contrast, or T1-weighted with contrast) where bounding box labels were placed on each image in which representative pathology or normal anatomical variant was identified^17,18^. As in knee examinations, effort was made to try to include all the pathology within the bounding box while limiting the normal surrounding anatomy. In some cases, the pathology or normal anatomic variant displayed within a given examination was so extensive or diffuse that a study-level label was used to characterize the relevant images or the entire exam inclusive of the finding (i.e., diffuse white matter disease). The study-level label, in these instances, replaced the use of a bounding box. If no relevant pathology was identified on a given examination, no labels were provided.

Note there are several limitations to this dataset that bear acknowledgement. First, while the annotators are subspecialist radiologists in practice at leading academic medical centers, the lack of multiple annotators/repeated annotations to determine inter-rater/intra-rater reliability metrics or ensure consensus agreement is a limitation and should be considered in the use of these labels. Further work may include multiple annotations by multiple readers to further refine the clinical labels applied in fastMRI+. Additionally, the fastMRI knee MRI raw dataset contained only coronally acquired series while the brain MRI dataset contained only axially acquired series, each in a variety of pulse sequences and coils. Most knee/brain pathologies that are visible in the non-coronal/non-axial planes are also visible in coronal/axial planes, though not as well seen or as well characterized. For instance, patellofemoral cartilage in the knee and optic neuritis in the brain. While sufficient for annotation, it is important to note that true diagnostic interpretation in MRI for the included pathologies typically demands multi-sequence and multi-planar images for clinically accurate interpretation. What is more, only binding boxes indicating knee and brain diseases were exported and reported in this work which may limit the research applications of this dataset. Full segmentation of structures would be more laborious and would be a potential subject of future work. Thus, the annotations provided by fastMRI+ may be incomplete. In the future, raw MRI datasets containing fully sampled multi-planar and multi-sequence data would enable optimal clinical annotation.

### Statistical analysis

Label distribution analysis was conducted for both knee and brain datasets showing detailed label descriptions at the same time. Table 1 shows annotation count and subject count for corresponding image-level knee labels. Note ‘Artifact’ is a study-level label for the entire study rather than a label of individual images. Table 2 shows annotation count and subject count for corresponding image-level brain labels. Table 3 shows subject count for corresponding subject-level brain labels. Note subject count was provided to show the prevalence of given pathology.

**Table 1:** Knee label summary.

Label	Annotation Count	Subject Count
**Meniscus**		
Meniscus Tear	5658	663
Displaced Meniscal Tissue	232	56
**Bones and Cartilage**		
Bone-Subchondral Edema	986	196
Bone Lesion	183	29
Bone-Fracture/Contusion/Dislocation	1060	119
Cartilage Full Thickness Loss/Defect	615	122
Cartilage Partial Thickness Loss/Defect	2985	588
**Ligaments**		
ACL High Grade Sprain	678	101
ACL Low-Mod Grade Sprain	765	153
MCL High Grade Sprain	11	4
MCL Low-Mod Grade Sprain	285	121
PCL High Grade Sprain	18	3
PCL Low-Mod Grade Sprain	142	40
LCL Complex High Grade Sprain	14	3
LCL Complex Low-Mod Grade Sprain	130	48
**Other**		
Joint Effusion	1311	142
Joint Bodies	38	11
Periarticular Cysts	864	161
Muscle Strain	65	11
Soft Tissue Lesion	90	10
Patellar Retinaculum High Grade Sprain	24	4
Artifact	/	13

*Artifact is study-level label.

**Table 2:** Brain image-level label summary.

Image Level Label	Annotation Count	Subject Count
Absent Septum Pellucidum	3	1
Craniectomy	32	4
Craniotomy	1025	99
Craniotomy with Cranioplasty	43	3
Dural Thickening	351	30
Edema	369	44
Encephalomalacia	161	18
Enlarged Ventricles	300	38
Extra-Axial Mass	104	11
Intraventricular Substance	8	1
Likely Cysts	17	5
Lacunar Infarct	113	32
Mass	380	46
Nonspecific Lesion	757	124
Nonspecific White Matter Lesion	1826	173
Normal Variant	73	21
Paranasal Sinus Opacification	40	8
Pineal Cyst	2	1
Possible Artifact	505	52
Posttreatment Change	1262	99
Resection Cavity	199	27

*Likely Cysts is applied to small lesions (approximately 1 cm or less in diameter) which are difficult to distinguish from parenchymal, simple parenchymal neuronal cyst, and prominent perivascular space.

**Table 3:** Brain study-level label summary.

Study Level Label	Subject Count
Global Ischemia	1
Small Vessel Chronic White Matter Ischemic Change	221
Motion Artifact	33
Possible Demyelinating Disease	2
Colpocephaly	2
White Matter Disease	2
Innumerable Bilateral Focal Brain Lesions	2
Extra-Axial Collection	9
Normal for Age	371

## Data Records

We created separate annotation files for the 1172 validation knee datasets and 1001 brain datasets, all based on the fastMRI source data^9^. The annotation files (knee.csv and brain.csv) can be accessed from both fastmri-plus Synapse repository^19^ and fastMRI-plus GitHub repository (https://github.com/microsoft/fastmri-plus) in CSV formats. Four CSV files are included in the ‘Annotations’ folder. File names of all radiologist-interpreted dataset are stored in knee_file_list.csv and brain_file_list.csv, respectively. Annotations are contained in knee.csv and brain.csv. In each annotation CSV file, the file names (i.e., column ‘Filename’) are aligned with the naming in the fastMRI dataset. For each annotation, file name, slice number, bounding box information, and disease label are provided. The bounding box information includes four parameters, x, y, width (pixel), and height (pixel), representing the x and y coordinates of the upper-left corner, the width and height of the bounding box. Unit of the bounding box parameters is ‘pixel’. Study-level labels are marked as ‘Yes’ in column ‘Study Level’ for slice 0 of the corresponding subjects with no specified bounding box information.

## Technical Validation

A board-certified radiologist with 10 years of experience reviewed the overall quality of the MRI image dataset prior to annotation and clinical evaluation was performed by two additional board-certified subspecialist radiologists. We cleaned and validated raw annotation files following instructions from MD.ai Documentation (https://docs.md.ai/). Creation and publication of fastMRI+ code repository followed standard practices with release of open-source software. Specifically, files with annotations and associated tools and scripts were managed source code control, continuous integration tests, and code/data reviews.

## Usage Notes

**Fig. 1:**
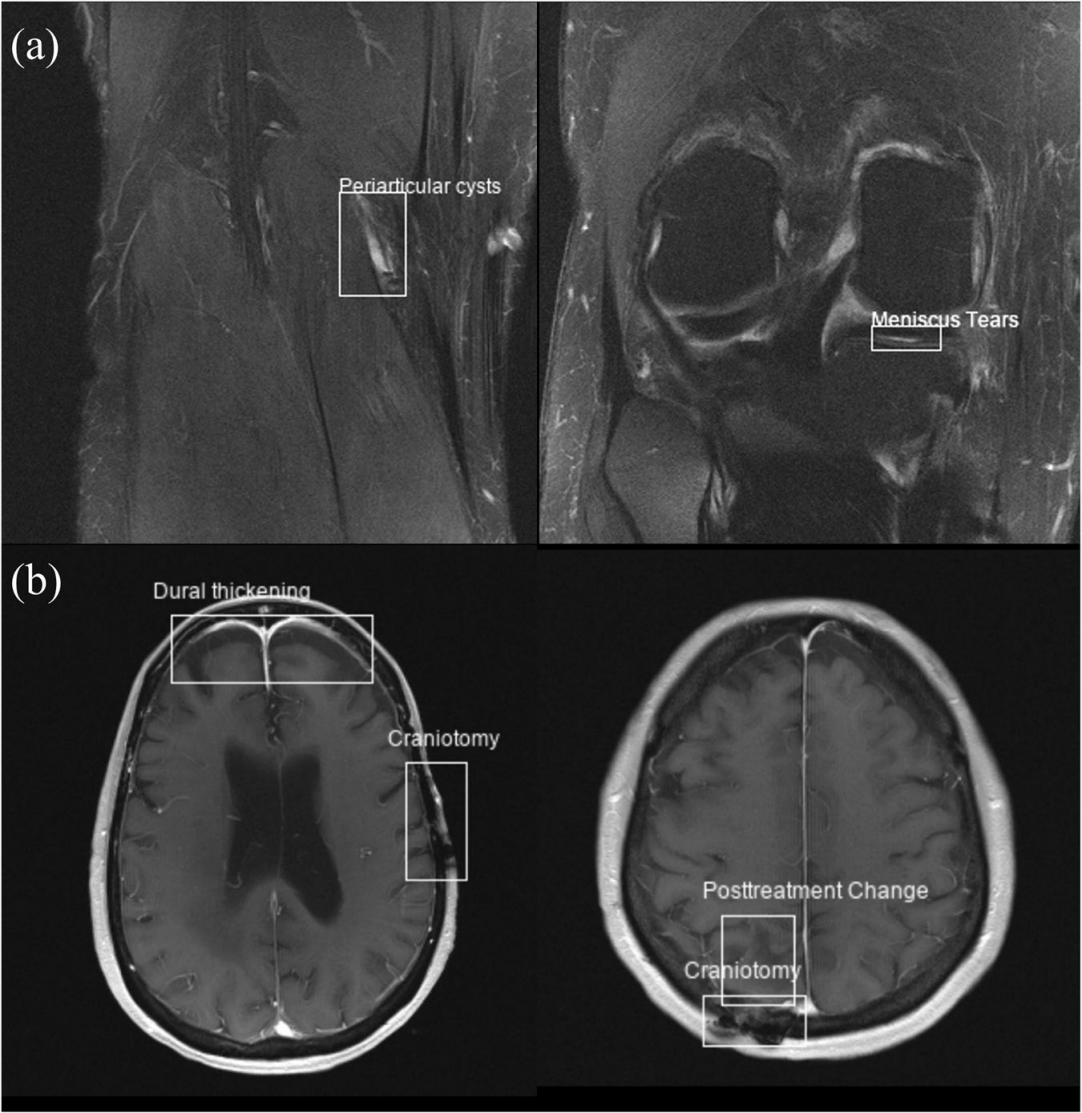
Example annotations (labels and bounding boxes) from the fastMRI+ dataset shown superimposed on both knee (**a**) and brain (**b**) reconstructed images from the fastMRI dataset.

The bounding box information can be used to plot overlaid bounding boxes on images, as shown in Fig. 1. The clinical labels, together with the bounding box coordinates, can also be converted to other formats (e.g., YOLO format^20^) in order to configure a classification or object detection problem. The open-source repository also contains an example Jupyter Notebook (‘ExampleScripts/example.ipynb’) of how to read the annotations and plot images with bounding boxes in Python.

## Data Availability

Scripts used to generate the DICOM images for radiologists can be accessed from (‘ExampleScripts/fastmri-to-dicom.py’) in the open-source GitHub repository. The detailed method used has been specified in the Methods section. More open-source tools for reconstructing the original fastMRI dataset, including standardized evaluation criteria, standardized code, and PyTorch data loaders can be found in the fastMRI GitHub repository (https://github.com/facebookresearch/fastMRI).
